# Deep sequencing of candidate genes identified 14 variants associated with smoking abstinence in an ethnically diverse sample

**DOI:** 10.1038/s41598-024-56750-7

**Published:** 2024-03-16

**Authors:** Paul M. Cinciripini, David W. Wetter, Jian Wang, Robert Yu, George Kypriotakis, Tapsi Kumar, Jason D. Robinson, Yong Cui, Charles E. Green, Andrew W. Bergen, Thomas R. Kosten, Steven E. Scherer, Sanjay Shete

**Affiliations:** 1 Department of Behavioral Science, The University of Texas MD Anderson Cancer Center, Houston, TX 77030 USA; 2 Department of Department of Population Health Sciences, University of Utah and Huntsman Cancer Institute, Salt Lake City, Utah 84112 USA; 3 Department of Biostatistics, The University of Texas MD Anderson Cancer Center, Houston, TX 77030 USA; 4 Department of Pediatrics, The University of Texas Medical School at Houston, Houston, TX 77030 USA; 5 Oregon Research Institute, Springfield, OR 97477 USA; 6 Department of Psychiatry, Baylor College of Medicine, Houston, TX 77030 USA; 7 Department of Molecular and Human Genetics, Human Genome Sequencing Center, Baylor College of Medicine, Houston, TX 77030 USA; 8 Department of Epidemiology, The University of Texas MD Anderson Cancer Center, Houston, TX 77030 USA

**Keywords:** Smoking cessation, Candidate gene study, Single-nucleotide polymorphism, Rare variant, Sequencing, Genetics, Behavioural genetics

## Abstract

Despite the large public health toll of smoking, genetic studies of smoking cessation have been limited with few discoveries of risk or protective loci. We investigated common and rare variant associations with success in quitting smoking using a cohort from 8 randomized controlled trials involving 2231 participants and a total of 10,020 common and 24,147 rare variants. We identified 14 novel markers including 6 mapping to genes previously related to psychiatric and substance use disorders, 4 of which were protective (*CYP2B6* (rs1175607105), *HTR3B* (rs1413172952; rs1204720503), rs80210037 on chr15), and 2 of which were associated with reduced cessation (*PARP15* (rs2173763), *SCL18A2* (rs363222)). The others mapped to areas associated with cancer including *FOXP1* (rs1288980) and *ZEB1* (rs7349). Network analysis identified significant canonical pathways for the serotonin receptor signaling pathway, nicotine and bupropion metabolism, and several related to tumor suppression. Two novel markers (rs6749438; rs6718083) on chr2 are flanked by genes associated with regulation of bodyweight. The identification of novel loci in this study can provide new targets of pharmacotherapy and inform efforts to develop personalized treatments based on genetic profiles.

## Introduction

Smoking is a well-established primary risk factor for several types of cancer^1^, cardiac disease^2^ and many other chronic illnesses^3^. It is responsible for nearly 8 million premature deaths each year worldwide (including 1.2 million deaths from second-hand smoke)^4^, and is the cause of substantial loss in productivity and increased healthcare expenditures in the US^5^. Several studies^6–12^ have shown that multiple aspects of smoking behavior are moderately heritable (~ 50%), including smoking cessation (~ 54%)^11^, and that this relationship may have increased over time^13^.

The overwhelming majority of studies relating genetic factors to smoking behavior have utilized large-scale epidemiological cross sectional and cohort samples and have concentrated on behavioral phenotypes that can be readily assessed through questionnaires and single item surveys, such as nicotine dependence, cigarettes per day, heaviness of smoking index, age of initiation and quitting status (current/former smoker). Multiple studies of this type has shown that non-overlapping SNPs from chr15q25.1, within the *CHRNA3*-*CHRNA5* -*CHRNB4* (nicotinic acetylcholine receptor) gene cluster, are consistently related to nicotine dependence^14–16^, with rs16969968 (within *CHRNA5*) having substantial influence, and a second signal tagged by rs680244 (*CHRNA3*)^17^. *CHRNA3* SNP rs1051730 also shows some of the strongest associations with nicotine dependence (cigarettes per day)^18^, as do the intronic SNPs rs588765 and rs578776, all of which are highly correlated with rs16969968^14,19,20^. The rs578776 SNP has demonstrated a protective effect in relation to nicotine dependence (minor allele more frequent in controls than dependent smokers). Joint analyses of rs16969968 and rs3743078 (highly correlated with rs578776) representing the risk and protective haplotypes at the cluster, resulted in a 2.4-fold increase in risk of heavy versus light smoking^21^. Other SNPs in this region have demonstrated nicotine dependence susceptible and protective haplotypes, and the relationship of these loci with heaviness of smoking is supported in meta-analysis of 34 datasets^20^**.** Another meta-analysis, involving 38,602 smokers with European and African origins across 15 studies, re-confirmed the association between smoking and SNPs in this gene cluster but also found that the SNP rs910083 C allele in the DNA methyltransferase 3 beta gene *DNMT3B* was associated with increased risk of nicotine dependence^22^. Other cohort studies have identified genetic markers of: 1) tobacco use and nicotine dependence^23–28^ including several from a cross-ancestry analysis of smokers of European and African descent (rs16969968 at *CHRNA5*, rs13284520 at *DBH*, rs151176846 at *CHRNA4*, rs2714700b between *MAGI2* and *GNAI1*, rs1862416 at *TENM2*)^29^ and of European and Asian decent (rs6474414 at *CHRNB3* and rs1072003 at CHRNA6); 2) number of self-reported quit-attempts, including SNPs rs6298, rs834829 and rs8192729 from *HTR1B*, *NR4A2*, and *CYP2A6* respectively^30^; 3) other addictive behaviors, such as alcohol use^10,29,31^; and 4) psychiatric disorders^29,32^. Most recently, in a large sample of both current and former smokers (~ 110K) and never smokers (~ 375K), an exome-wide association study (ExWAS) showed that rare predicted loss-of-function and likely deleterious missense variants in *CHRNB2* in aggregate were associated with a 35% decreased odds (protective) for smoking more than 10 cigarettes per day. An independent common variant of *CHRNB2*, rs2072659, also showed a protective effect for heavy smoking^33^.

GWAS studies of nicotine metabolism and clearance in European ancestry cohorts have shown significant associations between SNPs on chromosome 19 (including *CYP2A6*, *MAP3K10*, *ADCK4*, and *CYP2B6*) and on chromosome 4 (including *TMPRSS11E*) and the nicotine metabolism ratio (*trans*-3’-hydroxycotinine/cotinine or NMR) and between SNPs on chromosomes 9, 4 and 15 (including *CHRNB4*, *CHRNA3*, *and CHRNA5*) and measures of nicotine clearance (cotinine and the sum of cotinine and *trans*-3’-hydroxcotinine) ^34,35^. GWAS analysis in smokers of African American ancestry identified multiple independent SNPs at the *CYP2A6* chromosome 19 locus and two SNPs on chromosome 2 associated with the NMR; most of these SNPs were not previously identified in European ancestry cohorts.

While important for understanding population level associations between genetics and smoking, the type of studies noted above do not directly assess genetic factors that may drive quitting success during an actual quit attempt, as a smoker makes the transition from smoking to abstinence. The genetic mechanisms underlying the process of smoking cessation and relapse are poorly understood^36^. National surveys indicate nearly 70% of smokers want to quit smoking^37^, but despite over 50% of smokers attempting to quit each year, only about 7.5% achieve success annually^38^. Genetic studies that use a prospective sample of smokers trying to quit may more directly address biological substrates associated with cessation success and help improve treatment outcomes by providing new targets for pharmacotherapy and/or informing efforts at precision medicine that attempt to assign treatments to smokers based on a genetic profile^39^.

Although fewer in number, prospective studies of smokers attempting to quit have shown some promise in realizing these goals. For example, haplotypes of rs16969968 (*CHRNA5*) and rs680244 (*CHRNA3*) have demonstrated an association with abstinence among smokers receiving a placebo vs active pharmacotherapy for smoking cessation^40^**.** Moreover, an analysis of eight clinical trials^41^ found that minor alleles of *CHRNA3* rs1051730 and *CHRNA5* rs588765 were associated with increased abstinence among smokers receiving nicotine replacement therapy (NRT) but reduced abstinence among those receiving placebo, though these findings were not replicated in later studies^42,43^. The *CHRNB2* SNP rs2072661 has been associated with reduced cessation and the tryptophan 2,3-dioxygease (*TDO2*) SNP rs10517626 with enhanced cessation in a trial including NRT, placebo and bupropion, with the most pronounced effect of rs2072661 on the bupropion treated smokers^44^. Similarly in a clinical trial involving varenicline, bupropion or placebo, King and colleagues^45^ found that *CHRNB2* SNPs, most notably rs3811450, and SNPs in the *CHRNA5-CHRNA3-CHRNB4* region, e.g., rs7164594, were associated with increased abstinence among varenicline treated smokers; several SNPs from *CYP2B6*, including rs8109525, were associated with an enhanced response to bupropion specifically, and to overall cessation among all treated smokers.

In a first of its kind genetically informed treatment trial, Chen and colleagues^46^ examined the treatment response to combined NRT (patch plus lozenge) vs varenicline among smokers stratified by the *CHRNA5* SNP rs16969968 (GG vs. AA/GA alleles) at treatment onset. Results showed that among African American smokers, compared with placebo, those with the GG genotype quit significantly more often with NRT but not varenicline, while those with the AA/GA genotype quit significantly more often with varenicline and not NRT. No treatment by genotype interactions were observed for European descent smokers. This group also observed that polygenic risk scores for age of smoking initiation (older) and smoking persistence (past failed attempts) were predictive of abstinence across two prospective treatment trials, though treatment specific interactions were not reported.

In another pioneering trial Lerman and colleagues^47^ randomly assigned smokers to patch NRT, varenicline or placebo, stratifying by the NMR, and showed that smokers classified as normal metabolizers were more likely to quit using varenicline vs. NRT, while slow metabolizers quit equally often on both medications. NMR is a genetically informed biomarker that encompasses multiple SNPs, particularly within *CYP2A6*.

While a highly desirable approach for addressing questions related to precision and development of pharmacotherapies, the limitation of studying genetic predictors of smoking cessation in prospective clinical trials is that such studies typically involve much smaller samples than the large-scale epidemiological studies noted above. Moreover, when multiple trials are combined to increase sample size, harmonization of measurements and time points across trials can be problematic. In the current study, we address these potential limitations by combining smoking cessation outcomes from a cohort of 2231 smokers across 8 smoking cessation studies, which shared several common instruments and measurement points and the baseline collection of DNA. To our knowledge, this is the largest prospective sample of its kind. In this paper, we present novel findings relating common and rare variants to cessation success at 6-month post-treatment follow-up, a commonly used standard for measuring long term treatment outcome^48^. This contrasts with several of the trials reviewed above that focused on abstinence at the end of treatment, typically 12 weeks. The availability of cessation data of all studies at the 6-month time-point and the measurement of abstinence using both self-report and biochemical verification enables us to examine the relationship between key smoking-related genes in an integrated, well-phenotyped, ethnically diverse sample and to evaluate the findings for these traits more systematically than has previously been possible. This study can significantly improve our understanding of the etiology and pathophysiology of this complex phenotype, and aid in prevention and treatment efforts.

## Methods

### Subjects

The study included smokers who participated in 7 NIH- and 1 CPRIT- (Cancer Prevention Institute of Texas) funded studies of smoking cessation awarded to Drs. Paul Cinciripini and David Wetter, conducted at the University of Texas MD Anderson Cancer Center. Details of the design, recruitment, and inclusion/exclusion criteria for each of the studies have been described in detail elsewhere and include: Breakfree^49^, CARE^50^, CASSI^51^, MIND^52^, PNS^53^, QuitRx^54^, STEPS^55^ and Two2Quit^56^ (Grant numbers and ClinicalTrials.gov registration numbers, where required, are provided in the acknowledgements). Participants were recruited from the Houston metropolitan area from a wide variety of sources including local print media, flyers, and collaborations with local healthcare institutions. All studies were prospective smoking cessation clinical trials which shared at a minimum, recruitment of current smokers wanting to quit, exposure to smoking cessation guideline based treatment^57^ involving behavioral counseling for smoking cessation and pharmacotherapy, common measures of abstinence and 6 month outcome information. Regardless of treatment type, 6-month post-treatment outcome was the primary outcome variable for this analysis. All participants signed an informed consent form that permitted us to collect buccal swab samples and demographic and phenotypic data shared across studies. The study protocols were approved by the Institutional Review Board of MD Anderson in accordance with tenets of the Declaration of Helsinki.

### Outcome of interest

Abstinence status was measured at the end of six months using the self-reported 7-day point prevalence (no smoking even a puff in last 7 days) verified by exhaled carbon monoxide (CO) at or below a cutoff of 4 parts per million (ppm). Such a cutoff was recommended to verify smoking abstinence and has been shown to be more accurate than cutoffs of 8 or 10 ppm^58–60^. Based on the affirmative self-report of no smoking in the last 7 days plus CO ≤ 4 ppm, participants were classified as abstainers (i.e., successfully quit smoking). Individuals who reported smoking or had a CO > 4 ppm were classified as nonabstainers.

### Covariates

Demographic information (age, gender, race/ethnicity), type of cessation treatment received, and baseline smoking information (e.g., numbers of cigarettes smoked per day), were used as covariates in the analyses. The participants were treated using different medications for smoking cessation, including bupropion, nortriptyline, varenicline, NRT, combination of varenicline and bupropion, and placebo. Because the smoking cessation counseling duration and the pharmacotherapies differed across some of the trials, a study ID and a designator for medication type were included in all analyses as covariates. In addition, covariates for population structure, as described below, were included in the model.

### Sequencing and genotyping

The final sample consisted of 2231 participants prospectively enrolled in smoking cessation trials across the 8 studies shown in Table 1, for which both genetic and phenotypic (abstinence) information was available. For each of these individuals we collected DNA samples using buccal swabs, a 30″ mouth rinse using standard travel size bottle of Scope mouthwash (~ 2.5 oz) and processed using genomic DNA purification kits from Qiagen. The sample included a total of 10,020 genetic markers that were derived from both sequencing and genotyping procedures as described below.

**Table 1:** Distributions of population characteristics in the two-phase study (N = 2231).

	Phase 1					Phase 2				
Variable	*N*	Non-Abstainers	%	Abstainers	%	*N*	Non-Abstainers	%	Abstainers	%
**Age**	1571					660				
Median (Range)		44 (18–74)		46 (21–73)			44 (20–69)		45 (20–65)	
Mean (SD)		43.3 (10.9)		45.7 (11.4)			43.3 (10.9)		44.4 (10.2)	
**Cigarette Per Day**	1569					660				
Median (Range)		20 (3–91)		20 (5–60)			20 (5–80)		20 (5–80)	
Mean (SD)		20.8 (10.2)		19.0 (9.1)			20.7 (10.2)		19.9 (10.1)	
**Sex**	1571	1296		275		660	540		120	
Male	825	671	51.8	154	56.0	351	287	53.1	64	53.3
Female	746	625	48.2	121	44.0	309	253	46.9	56	46.7
**Study Name**	1571	1296		275		660	540		120	
Breakfree	264	247	19.1	17	6.2	99	92	17.0	7	5.8
CARE	227	214	16.5	13	4.7	94	88	16.3	6	5.0
CASSI	348	262	20.2	86	31.3	170	126	23.3	44	36.7
MIND	94	84	6.5	10	3.6	40	33	6.1	7	5.8
PNS	132	108	8.3	24	8.7	47	37	6.9	10	8.3
QuitRx	234	178	13.7	56	20.4	94	73	13.5	21	17.5
STEPS	34	27	2.1	7	2.5	15	14	2.6	1	0.8
Two2Quit	238	176	13.6	62	22.5	101	77	14.3	24	20.0
**Employment**	1548	1277		271		649	531		118	
Employed	1027	812	63.6	215	79.3	427	336	63.3	91	77.1
Unemployed	521	465	36.4	56	20.7	222	195	36.7	27	22.9
**Medical Treatment**	1571	1296		275		660	540		120	
Bupropion	77	57	4.4	20	7.3	30	20	3.7	10	8.3
Varenicline	164	118	9.1	46	16.7	76	59	10.9	17	14.2
Varenicline + Bupropion	101	72	5.6	29	10.5	44	34	6.3	10	8.3
Nortriptyline	7	4	0.3	3	1.1	10	9	1.7	1	0.8
Nicotine replacement (NRT)	1099	942	72.7	157	57.1	465	390	72.2	75	62.5
Placebo	123	103	7.9	20	7.3	35	28	5.2	7	5.8
**Education**	1559	1287		272		654	535		119	
High school/GED or less	551	476	37.0	75	27.6	220	192	35.9	28	23.5
Some college or associate degree	699	581	45.1	118	43.4	298	243	45.4	55	46.2
Bachelor degree	216	162	12.6	54	19.9	90	68	12.7	22	18.5
Some post-graduate work or above	93	68	5.3	25	9.2	46	32	6.0	14	11.8
**Race/Ethnicity**	1571	1296		275		660	540		120	
White,Hispanic	211	169	13.0	42	15.3	75	61	11.3	14	11.7
White,Non-Hispanic	678	525	40.5	153	55.6	298	234	43.3	64	53.3
Black,Hispanic	2	1	0.1	1	0.4	2	1	0.2	1	0.8
Black,Non-Hispanic	597	531	41.0	66	24.0	238	206	38.1	32	26.7
Black,Other	2	1	0.1	1	0.4	0	0	0.0	0	0.0
Other,Hispanic	45	40	3.1	5	1.8	25	22	4.1	3	2.5
Other,Non-Hispanic	36	29	2.2	7	2.5	22	16	3.0	6	5.0

SD: standard deviation.

Sequencing was carried out using the Illumina Hiseq2000 sequencing system at the Human Genome Sequencing Center, Baylor College of Medicine. We sequenced 55 candidate genes (Supplementary Material Table S1) primarily covering exon regions. Candidate genes were selected based upon literature survey of markers previously associated with smoking phenotypes (including dependence and cessation), other substance abuse and psychiatric disorders. The short sequence reads were filtered by Illumina CASAVA analysis software (v 13.10.01) and mapped to the reference genome using Burrows-Wheeler Aligner (BWA)^61^ to create a .bam file. Variants were called by Atlas-SNP2 (v1.4.3 r171, includes Atlas-Indel)^62^ to create a VCF file and these in turn were annotated using the Cassandra pipeline^63^. The average coverage of the target bases for the samples was 221x. Standard quality assurance and quality control procedures were conducted to detect problems with initial DNA quality, library construction methods, emulsion and bead quality, instrument chemistry and performance during the run and final sequence metrics after completion of the run. The initial result of the sequencing identified a total of 45,365 variants. Those not having a quality score of “PASS”, lower genotyping rate (< 0.95) and failing the Hardy–Weinberg proportion (HWP) test (*p* < 10^–6^) were removed, resulting in a total of 5138 common variants with a minor allele frequency (MAF) ≥ 0.05 and 24,197 rare variants (analyzed separately) with a MAF < 0.01 but ≥ 0.0001.

For the genotyping, we used Illumina’s Infinium iSelect Custom Genotyping Chip that included 6839 tagging SNPs. The tagging SNPs selected for this analysis, were derived from the literature identifying genetic markers associated with tobacco use, substance abuse and psychiatric disorders, plus those derived from our sequenced SNPs and included 169 ancestral informative markers. A set of 75 duplicate samples were genotyped to ensure genotyping quality. Illumina GenomeStudio was used for genotype calling based on the GenTrain clustering algorithm^64^. Cluster boundaries were determined using samples from the study. SNPs were filtered according to GenCall Score (GC score) > 0.15 and a median score of 0.88 using GenomeStudio (v1.9.4). Furthermore, we removed SNPs with a MAF < 0.05, and those that failed HWP test (*P* value < 10^–6^). The Genome Reference Consortium Human Build 37 (GRCh37) was used to map the genetic variants.

PLINK^65^ software (v1.90b3) was used to convert sequencing VCF and GenomeStudio files, and to process basic quality controls. The final analytic sample following quality control and availability of 6-month smoking cessation outcome data (the phenotypic of interest here) was 2231, which included 5138 variants from sequencing and 4882 additional markers from genotyping for a total of 10,020 markers used in the analyses (see Supplementary Information 2). The final composition of genetic information for the 2231 participants used in this analysis, included 1169 that had both sequencing and genotyping data, 439 and 623 with sequencing or genotyping alone, respectively. The mix of subjects in the datasets of two phases were proportional across these subsets.

### Population structure

Population structure was assessed using the Structure (v.2.3.4) program^66^. For assurance of the results, analysis using Admixture (v.1.3.0)^67,68^ was done on the same data sets. Population reference data from 1000 Genome Project (Phase 3 release, 2504 individuals)^69^ were used. The reference data contains 2478 unrelated individuals and over 84 million SNPs from 5 major racial groups from 26 geographic locations, African (7 locations), Latin American (4 locations), European (5 locations), East Asian (5 locations), and South Asian (5 locations)^70^. We first extracted 5317 markers with a minor allele frequency (MAF) ≥ 0.05 from the reference data set, which were overlapping with our data, and had a *P* value ≥ 1 × 10^–6^ for the HWP test. A general measurement of informativeness for assignment^71^ was calculated for each of the 5317 markers using the reference data. A set of 935 SNPs (including 169 ancestry informative markers) with a measurement of informativeness > 0.05 were selected for assessing population structure in our study.

The number of ancestries (*K*) was estimated with the use of the admixture model, based on the set of 935 SNPs. We considered a range of 5 to 15 for the number of ancestries *K*. For each of the value’s *K*, the Markov chain Monte Carlo (MCMC) process ran for 15,000 iterations, among which the first 5000 iterations were used as burn-in process. The likelihoods of the data given different *K* values (i.e., posterior probabilities) were calculated and the *K* value which maximized the posterior probability was selected as the number of ancestries in the study population. We thus obtained *K* = 11 ancestries for our study.

Given 11 ancestries, for each individual, STRUCTURE provided the probability of this individual belonging to each of the ancestry group (i.e., 11 probabilities per individual). One can assign the individual to one of the ancestries based on the highest probability. In our study, we created the population cluster score for each individual based on his/her ancestries corresponding to the three highest probabilities, which provided a higher resolution to classify individuals into different ancestries. The population structure scores created in this way were included in the statistical analysis as a covariate.

### Statistical analyses

Statistical analyses were conducted using PLINK^65^ (v1.90), SKAT-O^72,73,^ R^74^, SAS 9.3 (SAS Institute, Cary NC) and KING^75^ (v1.4) software. We used the genotypic data to identify individuals with discordant gender information, duplicates, and closely related individuals. We identified genetically related individuals by estimating the pairwise kinship coefficients using KING (v1.4) software. For any pair of individuals which were duplicates or related (i.e., with allele sharing of > 80%), we excluded the individual with lower call rate. Deviation from HWP for each genetic variant was assessed by 1 degree-of-freedom *χ*^*2*^ test or Fisher’s exact test where an expected cell count was less than five^76^.

Association analyses for common variants (SNPs; MAF ≥ 0.05) were conducted using multivariable unconditional logistic regression based on a two-sided Wald test implemented in the software PLINK^65^. We tested each common variant assuming an additive genetic model. Age, gender, study ID, medication type and population cluster were included in the analyses as covariates. For the smoking cessation (abstinence) phenotype, participants with missing information (14% of the sample) were imputed as “smoking” as it is the common practice in smoking cessation studies.

The study data were randomly divided into phase 1 data (70% of the participants) and phase 2 data (30% of the participants). For the joint analysis with pooled data from both phases, we included a fixed indicator as a covariate for the phases to control for the possible confounding effects of phases. We used the standard established threshold of genome-wide significance level of *P* value 5 × 10^–8^ to declare statistical significance.

For the association analyses of rare variants (MAF ≤ 0.01), we conducted the gene-based analysis using the optimal Sequence Kernel Association Test (SKAT-O)^72,73^ , which uses the collapse method to test the joint effect of multiple rare variants within a gene region on a phenotype. Same covariates, including age, sex, study ID, medication type, population cluster, and the indicator for phases (phase 1/phase 2), were included in the analyses as covariates. To account for multiple testing issues, we used the significance level of 9.1 × 10^–4^ (i.e., 0.05/55) for the gene-based rare-variants genetic association analysis.

### Ingenuity pathway analysis

Ingenuity Pathway Analysis (IPA; Ingenuity® Systems, www.ingenuity.com) ^77^ is a software program employed to connect molecules based on the scientific data in the Ingenuity Knowledge Base, including information on biological interactions and functional annotations^78^. In this study, we used IPA to further explore the biological mechanism/insight of the genes that harbor the genetic variants identified to be significantly associated with the abstinence phenotype in the association analysis. These genes of interest are denoted as focus genes in IPA. The IPA core analysis function was employed to determine biological functions, search for signaling and metabolic canonical pathways, and generate relevant molecular networks on the basis of the focus genes^79,80^. IPA creates the biological functions and canonical pathways from the literature, independent of focus genes. Specifically, IPA core analysis compares the focus genes with all build-in canonical pathways and biological functions in the IPA database and identifies the canonical pathways/biological functions, which include genes that overlap with the focus genes. The molecular network is constructed based on the focus genes and the connections in which they function, based on the main assumption that the biological function involves locally dense interactions. The details regarding the network generation algorithm have been described (summarized in the Supplementary Materials)^81,82^. Importantly, when generating a network, the iterative algorithm attempts to connect additional non-focus genes from its entire database to any of the genes which have already involved in the gene network (focus or non-focus genes) if such genes are more likely to have connections (i.e., biological relationships) with the network. As these non-focus genes are from a background consisting of all genes in the database, the resulting relevant networks may potentially identify additional genes that interact with the focus genes associated with abstinence. These additional genes emerge as potential candidate genes of interest for future investigations of abstinence. The resulting network also presents a bigger view of the genes likely to be interacting and directly or indirectly associated with abstinence. To evaluate the resulting functions, pathways, or networks, *P* values are calculated using a right-tailed Fisher's exact test, which measures the likelihood that the association between the set of focus genes and a given function/pathway/network is due to random chance^81,82^.

## Results

### Characteristics of study populations

The study included 1571 participants (275 abstainers and 1296 non-abstainers) in the phase 1 data; and 660 participants (120 abstainers and 540 non-abstainers) in the phase 2 data (Table 1). In the phase 1 dataset, the distributions of age and cigarettes smoked per day were very similar in the abstainers and non-abstainers: mean age 45.7 (standard deviation [SD] = 11.4) for the abstainers and 43.3 (SD = 10.9) for the non-abstainers; mean cigarettes smoked per day, 19.0 (SD = 9.1) for the abstainers and 20.8 (SD = 10.2) for the non-abstainers. Approximately half of the participants were male (56% for abstainers and 51.8% for non-abstainers). There were more White (both Hispanic and non-Hispanic) participants who were abstainers (70.9%) than non-abstainers (53.5%), while more Black (Hispanic and non-Hispanic) participants were non-abstainers (41.2%) than abstainers (24.8%). More participants were employed in the abstainer group (79.3%) compared with the non-abstainer group (63.6%). More participants had a high school/GED or less education in the non-abstainers (37%); while more participants had a bachelor’s degree or some post-graduate work or above in the abstainers (29.1%). The majority of the participants were treated using NRT for smoking cessation (57.1% for abstainers and 72.7% for non-abstainers).

The population characteristics in the phase 2 dataset were very similar to those in the phase 1 dataset. The abstainer group had similar distributions of age (mean 44.4 [SD = 10.2]), cigarettes smoked per day (mean 19.9 [SD = 10.1]), and sex (53.3% male), compared with the non-abstainer group (mean age 43.3 [SD = 10.9], mean cigarettes per day 20.7 [SD = 10.2], 53.1% male; Table 1). Similarly, in the abstainers, more participants were Hispanic and non-Hispanic White (65%), employed (77.1%) and had a bachelor’s degree or some post-graduate work or above (30.3%). The majority of the participants were treated with NRT for smoking cession (62.5% for abstainers and 72.2% for non-abstainers).

### Analyses of common variants

We found 14 genetic variants associated with the abstinence phenotype that met the genome-wide statistical significance threshold (that is, *P* < 5 × 10^–08^; Table 2). A Manhattan plot for the joint analysis using data merged from both phases is shown in Supplementary Fig. S1.

**Table 2:** Summary for genetic variants associated with abstinence phenotype in the combined analysis.

CHR	SNP or variant	Gene*	Location (bp)	Major	Minor Allele		Phase 1		Phase 2		Combined	
				Allele	Allele	MAF	OR [95% CI]	*P*	OR [95% CI]	*P*	OR [95% CI]	*P*
19	rs1175607105	CYP2B6	41,520,210	C	T	0.13	2.11[1.56,2.84]	9.91 × 10^–07^	3.06[1.83,5.10]	1.81 × 10^–05^	2.34[1.83,2.98]	9.06 × 10^–12^
3	rs2173763	PARP15	122,329,160	A	G	0.26	0.63[0.54,0.73]	4.64 × 10^–10^	0.67[0.47,0.95]	2.41 × 10^–02^	0.64[0.55,0.74]	5.88 × 10^–10^
2	rs6749438	DNAJC27	25,190,127	G	A	0.16	0.55[0.44,0.69]	1.61 × 10^–07^	0.56[0.34,0.92]	2.25 × 10^–02^	0.55[0.45,0.67]	9.91 × 10^–10^
2	rs6718083	EFR3B	25,362,194	G	A	0.31	0.60[0.49,0.74]	3.12 × 10^–06^	0.52[0.36,0.76]	6.46 × 10^–04^	0.58[0.49,0.69]	1.82 × 10^–09^
10	rs7349	ZEB1	31,817,905	C	A	0.35	0.68[0.59,0.79]	5.65 × 10^–07^	0.70[0.53,0.94]	1.77 × 10^–02^	0.69[0.61,0.78]	2.28 × 10^–09^
5	rs6869603	–	150,142,849	T	G	0.30	0.66[0.56,0.76]	3.45 × 10^–08^	0.70[0.48,1.00]	4.97 × 10^–02^	0.67[0.58,0.76]	2.35 × 10^–09^
10	rs363222	SLC18A2	119,019,448	G	C	0.43	0.65[0.54,0.77]	7.74 × 10^–07^	0.74[0.55,1.01]	5.53 × 10^–02^	0.67[0.59,0.77]	3.73 × 10^–09^
11	rs1413172952	HTR3B	113,792,339	C	A	0.26	1.91[1.45,2.52]	3.74 × 10^–06^	2.16[1.33,3.51]	1.91 × 10^–03^	1.98[1.57,2.49]	6.10 × 10^–09^
3	rs1288980	FOXP1	71,105,863	A	G	0.31	0.65[0.54,0.77]	7.64 × 10^–07^	0.68[0.50,0.92]	1.19 × 10^–02^	0.65[0.57,0.76]	6.71 × 10^–09^
11	rs1204720503	HTR3B	113,781,550	C	T	0.16	2.05[1.42,2.96]	1.40 × 10^–04^	2.57[1.47,4.48]	9.12 × 10^–04^	2.17[1.65,2.85]	2.72 × 10^–08^
10	rs992528	–	118,162,582	T	T	0.37	0.72[0.64,0.81]	7.15 × 10^–08^	0.64[0.46,0.89]	8.56 × 10^–03^	0.70[0.61,0.79]	3.22 × 10^–08^
15	rs80210037	–	78,727,819	G	T	0.21	1.81[1.40,2.34]	6.85 × 10^–06^	2.18[1.32,3.59]	2.24 × 10^–03^	1.91[1.52,2.40]	3.49 × 10^–08^
12	rs11064432	USP5	6,968,741	C	G	0.33	0.71[0.62,0.82]	2.99 × 10^–06^	0.71[0.55,0.92]	1.03 × 10^–02^	0.71[0.63,0.80]	4.14 × 10^–08^
13	rs1333758	NALCN	101,897,883	T	A	0.23	0.61[0.48,0.78]	9.50 × 10^–05^	0.62[0.47,0.82]	8.69 × 10^–04^	0.61[0.51,0.73]	4.40 × 10^–08^

*Human annotation release 1.

**Figure 1:**
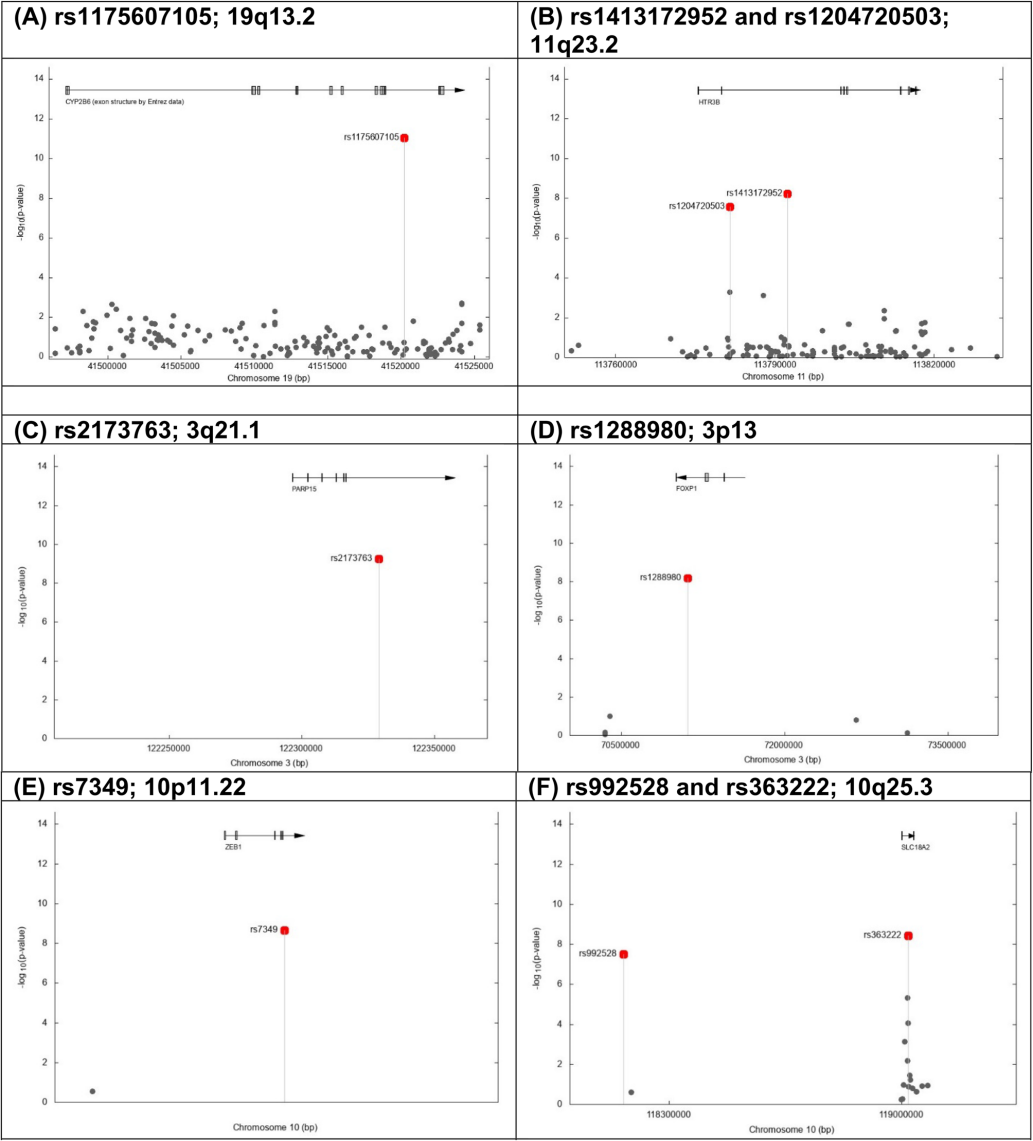

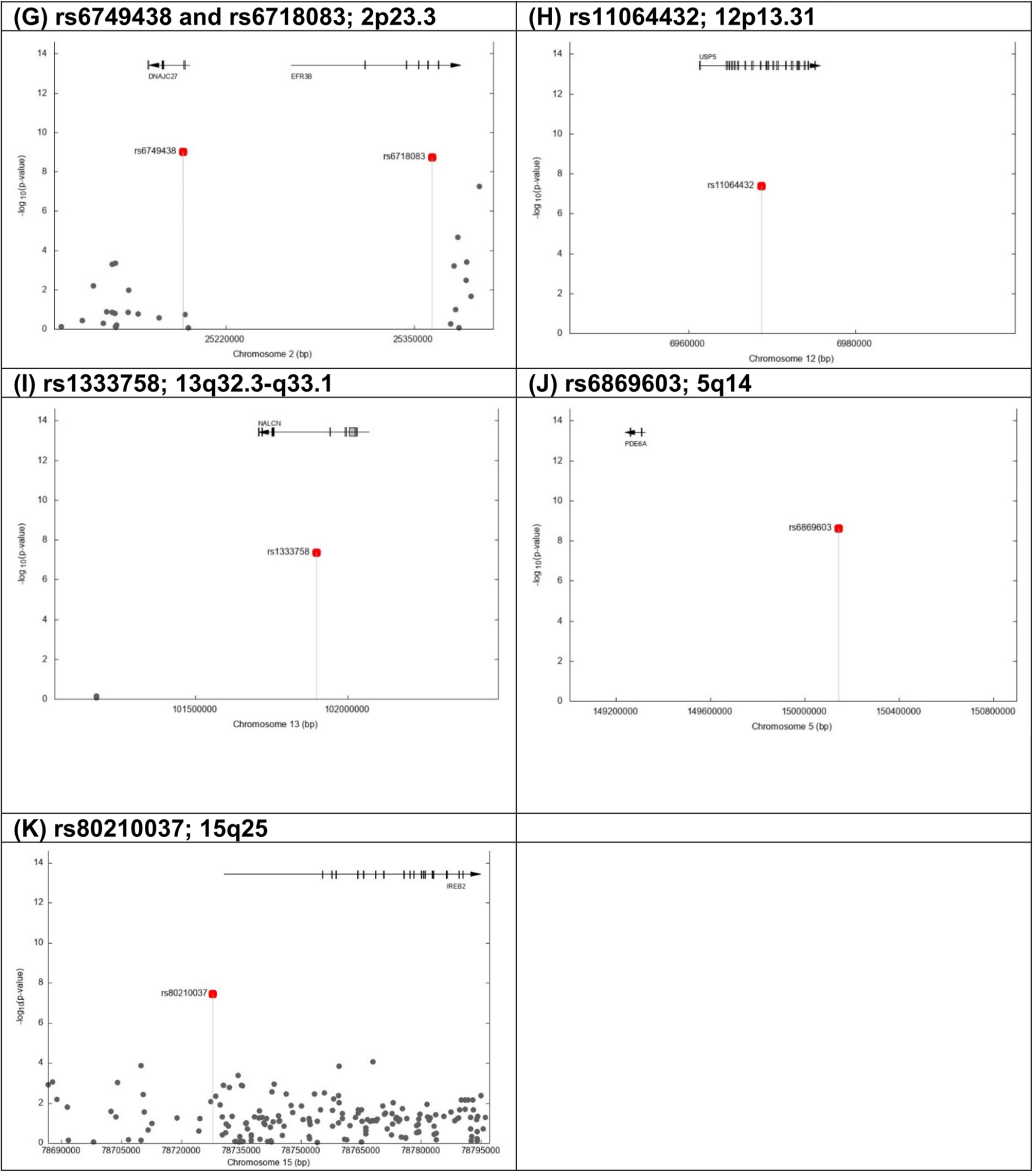
The genetic regions harboring the significant signals associated with smoking abstinence phenotype. y-axis: -log10(p-values) based on logistic regression. x-axis: base pair positions based on NCBI human annotation release 105. Grey dot: SNPs analyzed in the studies. Red dot: significant SNPs in the combined analysis.

Based on the *P* values using the meta-analysis of data from both phases, the variant rs1175607105 was found to be the strongest statistically significant signal protective for smoking cessation behavior (i.e., OR > 1; likely to quit smoking) (OR = 2.34, 95% CI: 1.83–2.98; *P* = 9.06 × 10^–12^). The rs1175607105 localizes to 19q13.2 (41,520,210 bp; Fig. 1A and Table 2) and maps to the gene *CYP2B6* (cytochrome P450 family 2 subfamily B member 6).

There were three additional genetic variants identified to be protective for smoking cessation behavior, including rs1413172952 (OR = 1.98, 95% CI: 1.57–2.49; *P* = 6.1 × 10^–9^), rs1204720503 (OR = 2.17, 95% CI: 1.65–2.85; *P* = 2.72 × 10^–8^) and rs80210037 (OR = 1.91, 95% CI: 1.52–2.40; *P* = 3.49 × 10^–8^). The variants rs1413172952 (113,792,339 bp; Fig. 1B and Table 2) and rs1204720503 (113,781,550 bp) localize to 11q23.2 and maps to the gene *HTR3B* (5-hydroxytryptamine receptor 3B).

In addition, ten risk genetic variants were identified as being significantly associated with the abstinence phenotype (i.e., OR < 1; less likely to quit smoking), including rs2173763 (OR = 0.64, 95% CI: 0.55–0.74; *P* = 5.88 × 10^–10^), rs6749438 (OR = 0.55, 95% CI: 0.45–0.67; *P* = 9.91 × 10^–10^), rs6718083 (OR = 0.58, 95% CI: 0.49–0.69; *P* = 1.82 × 10^–9^), rs7349 (OR = 0.69, 95% CI: 0.61–0.78; *P* = 2.28 × 10^–9^), rs6869603 (OR = 0.67, 95% CI: 0.58–0.76; *P* = 2.35 × 10^–9^), rs363222 (OR = 0.67, 95% CI: 0.59–0.77; *P* = 3.73 × 10^–9^), rs1288980 (OR = 0.65, 95% CI: 0.57–0.76; *P* = 6.71 × 10^–9^), rs992528 (OR = 0.70, 95% CI: 0.61–0.79; *P* = 3.22 × 10^–8^), rs11064432 (OR = 0.71, 95% CI: 0.63–0.80; *P* = 4.14 × 10^–8^) and rs1333758 (OR = 0.61, 95% CI: 0.51–0.73; *P* = 4.4 × 10^–8^).

Among the ten significant signals, two variants (rs2173763 and rs1288980) were located on chromosome 3. In particular, the variant rs2173763 localizes to 3q21.1 (122,329,160 bp; Fig. 1C and Table 2) and maps to the intron of the gene *PARP15* (poly(ADP-ribose) polymerase family member 15). The variant rs1288980 localizes to 3p13 (71,105,863 bp; Fig. 1D) and maps to the gene *FOXP1* (forkhead box P1).

Three variants (rs7349, rs363222, rs992528) were located on chromosome 10. The variant rs7349 localizes to 10p11.22 (31,817,905 bp; Fig. 1E) and maps to the 3’ untranslated region of the gene *ZEB1* (zinc finger E-box binding homeobox 1), which has been associated with lung cancer^83–85^. The variant rs363222 localizes to 10q25.3 (119,019,448 bp; Fig. 1F) and maps to the gene *SLC18A2* (solute carrier family 18 member A2).

Two variants (rs6749438 and rs6718083) were located on chromosome 2 and are close to each other. The variant rs6749438 localizes to 2p23.3 (25,190,127 bp; Fig. 1G) and maps to gene *DNAJC27* (DnaJ heat shock protein family (Hsp40) member C27). The variant rs6718083 localizes to 2p23.3 (25,362,194 bp; Fig. 1G) and maps to the gene *EFR3B* (EFR3 homolog B). The variant rs11064432 localizes to 12p13.31 (6,968,741 bp; Fig. 1H) and maps to the intron of the gene *USP5* (ubiquitin specific peptidase 5). The variant rs1333758 localizes to 13q32.3-q33.1 (101,897,883 bp; Fig. 1I) and maps to the gene *NALCN*, a gene that belongs to a family of voltage-gated sodium and calcium channels expressed throughout the nervous system^86^.

The linkage disequilibrium (LD) was assessed for the significant genetic variants (Table 2) in close proximity. No strong LD was observed between pairs of significant variants in proximity: rs6749438 and rs6718083 (${r}^{2}=0.07$), rs363222 and rs992528 (${r}^{2}=0.104$), and rs1413172952 and rs1204720503 (${r}^{2}=0.015$).

We further investigated the LD between the 14 significant genetic variants (Table 2) and the variants associated with smoking cessation from the literature. Specifically, we extracted SNPs associated with smoking cessation from the EBI/NHGRI GWAS Catalog^87^ as of January 30, 2024, selecting entries referencing "smoking cessation" as the disease/trait. We omitted SNPs from studies that compared current smokers to former smokers because such comparisons do not align with the methodology of our current study. Consequently, we identified three SNPs associated with smoking cessation^89,90^. We employed LDlink, a comprehensive web-based platform, to investigate the LD^92^. LDlink utilizes the genomic data from the 1000 Genomes Project, offering a rich repository of human genetic variation across diverse populations. The resulting ${r}^{2}$ values are reported in Supplementary Material Table S2. No strong LD was noted, with ${r}^{2}$ values ranging from < 0.001 to 0.027.

### Ingenuity pathway analysis (IPA)

From the analysis of common variants, we identified 10 genes that harbor the genetic variants significantly associated with smoking cessation (Table 2). The 10 identified genes were employed as the focus genes in the IPA core analysis. Significant canonical pathways and biological functions were identified based on the focus genes. As described in the Methods section, the core analysis provides a measure of the association of focus genes of interest with the built-in canonical pathways and biological functions. In particular, the most significant canonical pathway identified was the Serotonin Receptor signaling pathway (*P* = 1.78 × 10^–4^), which is relevant in the etiology of neuropsychiatric and mood disorders^88^. The focus genes were also shown to be potentially related to Bupropion Degradation and Nicotine Degradation pathways (*P* = 1.15 × 10^–2^ and 2.33 × 10^–2^, respectively), which are, in general, related to smoking cessation. The significant *P* values imply over-representation of focus genes in these pathways, and that the association between focus genes and pathways is non-random.

Furthermore, the IPA core analysis generated a network showing the additional molecules that directly or indirectly relate to or interact with the genes identified through the association analyses (Supplementary Figure S2). The molecules with most interconnections are of interest since the highly connected molecules are considered to be most likely associated with diseases or biological functions^79,81^. Fourteen molecules with 15 or more interactions, as indicated by the numbers of edges connected to other molecules in the network, were identified and are highlighted in the figure. The most highly connected molecules including several related to cancer etiology (see Discussion) such as: *TP53* (tumor protein p53)^91^; *RB1* (RB transcriptional corepressor)^93^; *CDKN2A* (cyclin dependent kinase inhibitor 2A)^94^ and *EGFR* (epidermal growth factor receptor)^95^, plus *RELA*, a subunit of the heterodimeric transcription factor called NF-Kappa-B, related to substance abuse^96^.

### Analyses of rare variants

Based on the gene-based association analyses for rare variants (MAF ≤ 0.01), we observed marginal associations of *ADCY5* (*P* = 1.16 × 10^–2^) and *SLC6A2* (*P* = 1.72 × 10^–2^) for smoking abstinence phenotype using data combined from both phases. Gene *ADCY5* localizes to 3q21.1 and has been associated with low birth weight and type 2 diabetes^97,98.^ Gene *SLC6A2* localizes to 16q12.2 which is associated with norepinephrine transport and bipolar disorder, depression and ADHD^99–101^. Note that these signals were not statistically significant after adjusting for multiple comparisons (a significance threshold of *P* value ≤ 9.1 × 10^–4^ corresponding to 0.05/55 candidate genes).

## Discussion

This study examined genetic predictors of long-term treatment success (6-months) for smoking cessation that used a prospective sample of 2331 smokers undergoing standard smoking cessation therapy including behavioral counseling and pharmacotherapy. Genotyping involved sequencing of 55 candidate genes previously associated with smoking behavior, other substance abuse and psychiatric disorders, and the 6606 tagging SNPs and 233 AIMS, yielding 10,020 common and 24,147 rare variants of sufficient quality for analysis. We took the approach of engaging in two phases using 70% and 30% of the sample, respectively and present the combined results of all markers exceeding GWAS defined significance levels, associating common and rare variants with the cessation phenotype, while controlling for multiple factors including genomic ancestry, study related factors, and demographics.

Our analysis revealed 14 novel markers not previously identified with smoking cessation phenotype of interest defined in the manuscript (*P* < 5 × 10^–08^). When mapping these SNPs to specific genes and regions, two major themes emerged. The first theme highlights shared genetic substrates between abstinence from smoking and selected psychiatric and substance use disorders among 6 of these markers, four of which were protective (OR > 1 favoring smoking cessation). Among them, the variant rs1175607105 produced the strongest signal and maps to the gene *CYP2B6* which is the primary enzyme responsible for metabolism of the smoking cessation and antidepressant drug bupropion^102,103^ but has also been implicated in nicotine metabolism^34,35,104,105^. While a modest inhibitor of norepinephrine and dopamine reuptake, which may account for its antidepressant effects, bupropion also acts as an antagonist of several nicotine cholinergic receptor subtypes^106^. The other 3 protective variants include rs1413172952, and rs1204720503 which map to the *HTR3B* (5-hydroxytryptamine-serotonin receptor 3B) gene and variant rs80210037 on chromosome 15. The *HTR3B* serotonergic receptor gene has been implicated in longer time to relapse following treatment in a combined analysis of bupropion, varenicline and placebo treated smokers^45^ and nicotine dependence in a mixed ancestry sample of African and European decent^107^. This suggests that loci on this gene may be predictive of smoking cessation treatment regardless of the type of pharmacotherapy given and of dependence on nicotine in a mixed ancestry sample. Interestingly, other polymorphisms on this gene have been related to a protective effect for obsessive compulsive disorder^108^ and major depression^109^ which, like other psychiatric disorders, have been associated with increased prevalence of smoking^110^ and a shared causal genetic basis^111,112^.

Two other novel variants, associated with reduced likelihood of quitting, also mapped to genes with previously noted markers for psychiatric and substance use disorders: rs2173763 maps to *PARP15* on which several locations have been associated with a broad mood disorder phenotype (Major Depression and Bipolar disorder)^113^; and rs363222 maps to *SLC18A2* which is associated with monoamine neurotransmitter transport (dopamine, norepinephrine, serotonin). Varenicline is one of our most effective smoking cessation medications^114^ and acts as a dopamine partial agonist^115^. Several other loci on this gene have been related to alcohol^116^, opioid^117^ and nicotine dependence^118,119^, and PTSD^120^. Moreover, while our analyses of rare variants did not yield significant associations that survived correction for multiple comparisons, a strong signal was present for *SLC6A2* (norepinephrine transporter) which has been implicated in mood disorders and ADHD^99–101^, both of which are more prevalent among smokers^110,121^.

Consistent with the relationship between smoking cessation and psychiatric disorders described above for individual markers mapped to specific genes, our IPA of the 10 significant common variants that mapped to specific genes (see Table 2) showed significant canonical pathways for the serotonin receptor signaling pathway and for nicotine and bupropion metabolism. Serotonin reuptake inhibitors are used routinely in the treatment of depression^122^, while as noted previously, differences in nicotine metabolism^47^ have been associated with a differential response to NRT, varenicline^47^, and bupropion^123^. Interestingly, the drug venlafaxine, a norepinephrine and serotonin reuptake inhibitor, has been associated with increased smoking cessation when combined with NRT^124^, which is commensurate with the findings noted above for *SLC18A2* and *SCL6A2*. Our IPA network analysis of related molecules also revealed relationship between these genes and * RELA*, a transcription factor involved in *NFkB* heterodimer formation, nuclear translocation and activation and previously implicated in drug addiction^96^.

The second theme among our results points to genetic regions associated with both smoking cessation and cancer pathophysiology (note smokers in this sample did not have a current cancer diagnosis, though past-history is unknown). For example, we found significant associations between abstinence and the variant rs1288980 mapping to the gene *FOXP1* containing regions reported to act as a tumor suppressor^125,126^. The variant rs7349 on chromosome 10 maps to the gene *ZEB1* which has been associated with invasiveness, metastasis and poor prognosis of lung cancer^83–85^. While previous studies have found associations between lung cancer^127–130^ and COPD^131,132^ and the *CHRNA5* SNP rs16969968, noted for its relationship to nicotine dependence^14^, our findings suggest that additional markers associated with poor cessation outcome may also be related to lung cancer pathophysiology.

These findings were extended by our IPA network analysis of molecules related to the genes identified above in our association analyses. The results further highlight the connection between genetic predictors of smoking cessation and cancer, most likely attributable to tobacco smoke exposure. Relations with several tumor suppressors were noted, including: *TP53*^91^ which is associated with tobacco related mutations^133,134^ and several cancer types including breast, leukemia, cervical^135–137^ and lung^133,138,139^; *RB1*, is related to several cancers including childhood retinoblastoma, osteogenic sarcoma, bladder^93^ and lung, specifically with regard to smoking behavior^140,141^; and *CDKN2A*, which has been associated with a wide variety of cancers,^94^ including those that are tobacco-related, such as head and neck squamous cell carcinoma, oral and lung cancer^142–145^. Numerous interactions were also noted for *MYC*, a proto-oncogene^146^ and *EGFR***.** Down regulation of c-Myc is associated with invasion/migration capacity of bronchial epithelial cells exposed to cigarette smoke extract^147^. *EGFR*^95^ mutations act an oncogenic driver of lung cancer in non-smokers and light smokers^148–150^.

Other findings include two variants, rs6749438 and rs6718083 mapped to genes *DNAJC27* and *EFR3B*, on chromosome 2, respectively. While there is no specific information for rs6749438, that region is flanked by *ERF3B* and *ADCY3*. Multiple studies have linked SNPs in this area to regulation of body weight, obesity and BMI^151,152^. Interactions between body weight and smoking have been reported for rs16969968-rs1051730 in the *CHRNA5-A3-B4* cluster which are associated with reduced bodyweight in smokers but increased body weight in nonsmokers^153^. Of the remaining variants, no relevant information is available for rs11064432 which maps to the intron of the gene *USP* or rs1333758 mapping to *NALCN*.

Finally, we did not find associations with rare variants that survived correction for multiple comparisons, although the strongest signal was noted for *SLC6A2*, associated with norepinephrine transport: a finding consistent with our other observations associating smoking cessation with regulation of monoamine neurotransmitters especially serotonergic as noted above.

## Conclusions

In this study of over 2000 smokers attempting to quit smoking from a multiple ancestry sample, we found 14 novel markers, not previously identified with smoking cessation. When mapped to specific genes and regions, shared genetic substrates between abstinence from smoking and selected psychiatric and substance use disorders were noted among 6 of these markers, four of which were protective. Strong signals were observed for *CYP2B6; HTR3B*; *PARP15*; *SCL18A2*; and *SLC6A2*. Loci within the *HTR3B* gene may be of particular interest as they may be predictive of smoking cessation regardless of the type of pharmacotherapy administered. Our network analysis also showed significant canonical pathways for the serotonin receptor signaling pathway and for nicotine and bupropion metabolism. We also found several markers of smoking cessation among genes previously implicated in the development of cancer. These included *FOXP1* and *ZEB1*; and through our network analysis, *TP53*; *RB1*; *CDKN2*; *MYC* and *EGFR*. Two novel markers (rs6749438; rs6718083) on chromosome 2 are flanked by genes associated with regulation of bodyweight. Overall, our results identified several novel genetic markers of smoking cessation, both protective and at-risk, both individually and in combination. Larger studies are needed to identify future targets for smoking cessation pharmacotherapy and personalized treatment based on genetic profiles.

### Supplementary Information

Supplementary Information 1.

Supplementary Information 2.

## Data Availability

The dataset generated and analyzed in this study is available from the corresponding author on a reasonable request.

## References

[1] US Department of Health and Human Services. *The Health Consequences of Smoking—50 Years of Progress: A Report of the Surgeon General.* Atlanta, GA: Centers for Disease Control and Prevention, National Center for Chronic Disease Prevention and Health Promotion, Office on Smoking and Health (2014).

[2] Ambrose JA, Barua RS (2004). The pathophysiology of cigarette smoking and cardiovascular disease. An update. J. Am. Coll. Cardiol..

[3] Yanbaeva DG, Dentener MA, Creutzberg EC, Wesseling G, Wouters EFM (2007). Systemic effects of smoking. Chest..

[4] World Health Organization. Global report on trends in prevalence of tobacco use 2000–2025. Accessed February 24 (2022).

[5] US Department of Health and Human Services. *Smoking Cessation: A Report of the Surgeon General.* Washington (DC) (2020).

[6] Kendler KS, Schmitt E, Aggen SH, Prescott CA (2008). Genetic and environmental influences on alcohol, caffeine, cannabis, and nicotine use from early adolescence to middle adulthood. Arch. Gen. Psychiatry..

[7] Kim Y-K (2010). Handbook of behavior genetics.

[8] Zeiger JS, Haberstick BC, Schlaepfer I (2008). The neuronal nicotinic receptor subunit genes (CHRNA6 and CHRNB3) are associated with subjective responses to tobacco. Hum. Mol. Gen..

[9] Haberstick BC, Zeiger JS, Corley RP (2011). Common and drug-specific genetic influences on subjective effects to alcohol, tobacco and marijuana use. Addiction..

[10] Liu M, Jiang Y, Wedow R (2019). Association studies of up to 1.2 million individuals yield new insights into the genetic etiology of tobacco and alcohol use. Nat. Genet..

[11] Xian H, Scherrer JF, Madden PAF (2003). The heritability of failed smoking cessation and nicotine withdrawal in twins who smoked and attempted to quit. Nicotine Tob. Res..

[12] Evans LM, Jang S, Hancock DB (2021). Genetic architecture of four smoking behaviors using partitioned SNP heritability. Addiction..

[13] Domingue BW, Conley D, Fletcher J, Boardman JD (2016). Cohort effects in the genetic influence on smoking. Behav. Genet..

[14] Bierut LJ, Stitzel JA, Wang JC (2008). Variants in nicotinic receptors and risk for nicotine dependence. Am. J. Psychiatry..

[15] Lee S-H, Ahn W-Y, Seweryn M, Sadee W (2018). Combined genetic influence of the nicotinic receptor gene cluster CHRNA5/A3/B4 on nicotine dependence. BMC Genom..

[16] Xu K, Li B, McGinnis KA (2020). Genome-wide association study of smoking trajectory and meta-analysis of smoking status in 842,000 individuals. Nat. Commun..

[17] Weiss RB, Baker TB, Cannon DS (2008). A candidate gene approach identifies the CHRNA5-A3-B4 region as a risk factor for age-dependent nicotine addiction. PLoS Genet..

[18] Pérez-Morales R, González-Zamora A, González-Delgado MF (2018). CHRNA3 rs1051730 and CHRNA5 rs16969968 polymorphisms are associated with heavy smoking, lung cancer, and chronic obstructive pulmonary disease in a mexican population. Ann. Hum. Genet..

[19] Agrawal A, Bierut LJ (2012). Identifying genetic variation for alcohol dependence. Alcohol Res..

[20] Saccone NL, Culverhouse RC, Schwantes-An TH (2010). Multiple independent loci at chromosome 15q25.1 affect smoking quantity: A meta-analysis and comparison with lung cancer and COPD. PLoS Genet..

[21] Stevens VL, Bierut LJ, Talbot JT (2008). Nicotinic receptor gene variants influence susceptibility to heavy smoking. Cancer Epidemiol. Biomarkers Prev..

[22] Hancock DB, Guo Y, Reginsson GW (2017). Genome-wide association study across European and African American ancestries identifies a SNP in DNMT3B contributing to nicotine dependence. Mol. Psychiatry..

[23] Tobacco and Genetics Consortium (2010). Genome-wide meta-analyses identify multiple loci associated with smoking behavior. Nat. Genet..

[24] Erzurumluoglu AM, Liu M, Jackson VE (2020). Meta-analysis of up to 622,409 individuals identifies 40 novel smoking behaviour associated genetic loci. Mol. Psychiatry..

[25] Loukola A, Wedenoja J, Keskitalo-Vuokko K (2014). Genome-wide association study on detailed profiles of smoking behavior and nicotine dependence in a twin sample. Mol. Psychiatry..

[26] Hancock DB, Wang J-C, Gaddis NC (2015). A multiancestry study identifies novel genetic associations with CHRNA5 methylation in human brain and risk of nicotine dependence. Hum. Mol. Genet..

[27] Saccone NL, Emery LS, Sofer T (2018). Genome-wide association study of heavy smoking and daily/nondaily smoking in the Hispanic community health study/Study of Latinos (HCHS/SOL). Nicotine Tob. Res..

[28] Wessel J, McDonald SM, Hinds DA (2010). Resequencing of nicotinic acetylcholine receptor genes and association of common and rare variants with the Fagerström test for nicotine dependence. Neuropsychopharmacol..

[29] Quach BC, Bray MJ, Gaddis NC (2020). Expanding the genetic architecture of nicotine dependence and its shared genetics with multiple traits. Nat. Commun..

[30] Stevens VL, Jacobs EJ, Gapstur SM (2017). Evaluation of a novel difficulty of smoking cessation phenotype based on number of quit attempts. Nicotine Tob. Res..

[31] Matoba N, Akiyama M, Ishigaki K (2020). GWAS of 165,084 Japanese individuals identified nine loci associated with dietary habits. Nat. Hum. Behav..

[32] Taylor AE, Fluharty ME, Bjørngaard JH (2014). Investigating the possible causal association of smoking with depression and anxiety using Mendelian randomisation meta-analysis. The CARTA consortium. BMJ Open..

[33] Rajagopal VM, Watanabe K, Mbatchou J (2022). Rare coding variants in CHRNB2 reduce the likelihood of smoking. medRxiv.

[34] Buchwald J, Chenoweth MJ, Palviainen T (2021). Genome-wide association meta-analysis of nicotine metabolism and cigarette consumption measures in smokers of European descent. Mol. Psychiatry..

[35] Chenoweth MJ, Ware JJ, Zhu AZX (2018). Genome-wide association study of a nicotine metabolism biomarker in African American smokers. Impact of chromosome 19 genetic influences. Addiction..

[36] Hancock DB, Markunas CA, Bierut LJ, Johnson EO (2018). Human genetics of addiction. New insights and future directions. Curr. Psychiatry Rep..

[37] Babb S, Malarcher A, Schauer G, Asman K, Jamal A (2017). Quitting smoking among adults—United States, 2000-2015. MMWR Morb. Mortal. Wkly. Rep..

[38] Creamer MR, Wang TW, Babb S (2019). Tobacco product use and cessation indicators among adults—United States, 2018. MMWR Morb. Mortal. Wkly. Rep..

[39] Chen L-S, Zawertailo L, Piasecki TM (2018). Leveraging genomic data in smoking cessation trials in the era of precision medicine. Why and How. Nicotine Tob. Res..

[40] Chen LS, Baker TB, Piper ME (2012). Interplay of genetic risk factors (CHRNA5-CHRNA3-CHRNB4) and cessation treatments in smoking cessation success. Am. J. Psychiatry..

[41] Bergen AW, Javitz HS, Krasnow R (2013). Nicotinic acetylcholine receptor variation and response to smoking cessation therapies. Pharmacogenet. Genom..

[42] Leung T, Bergen A, Munafò MR, de Ruyck K, Selby P, de Luca V (2015). Effect of the rs1051730–rs16969968 variant and smoking cessation treatment. A meta-analysis. Pharmacogenomics..

[43] Tyndale RF, Zhu AZX, George TP (2015). Lack of associations of CHRNA5-A3-B4 genetic variants with smoking cessation treatment outcomes in caucasian smokers despite associations with baseline smoking. PloS one..

[44] Conti DV, Lee W, Li D (2008). Nicotinic acetylcholine receptor beta2 subunit gene implicated in a systems-based candidate gene study of smoking cessation. Hum. Mol. Genet..

[45] King DP, Paciga S, Pickering E (2012). Smoking cessation pharmacogenetics: analysis of varenicline and bupropion in placebo-controlled clinical trials. Neuropsychopharmacol..

[46] Chen L-S, Baker TB, Miller JP (2020). Genetic variant in CHRNA5 and response to varenicline and combination nicotine replacement in a randomized placebo-controlled trial. Clin. Pharmacol. Ther..

[47] Lerman C, Schnoll RA, Hawk LW, JR, (2015). Use of the nicotine metabolite ratio as a genetically informed biomarker of response to nicotine patch or varenicline for smoking cessation: A randomised, double-blind placebo-controlled trial. Lancet Respir. Med..

[48] Piper ME, Bullen C, Krishnan-Sarin S (2020). Defining and measuring abstinence in clinical trials of smoking cessation interventions. An updated review. Nicotine Tob. Res..

[49] Heppner WL, Spears CA, Correa-Fernandez V (2016). Dispositional mindfulness predicts enhanced smoking cessation and smoking lapse recovery. Ann. Behav. Med..

[50] Cambron C, Haslam AK, Baucom BRW (2019). Momentary precipitants connecting stress and smoking lapse during a quit attempt. Health. Psychol..

[51] Cinciripini PM, Minnix JA, Robinson JD (2023). The effects of scheduled smoking reduction and Precessation nicotine replacement therapy on smoking cessation. Randomized controlled trial with compliance. JMIR Form. Res..

[52] Spears CA, Hedeker D, Li L (2017). Mechanisms underlying mindfulness-based addiction treatment versus cognitive behavioral therapy and usual care for smoking cessation. J. Consult. Clin. Psychol..

[53] Haslam AK, Correa-Fernández V, Hoover DS, Li L, Lam C, Wetter DW (2018). Anhedonia and smoking cessation among Spanish-speaking Mexican-Americans. Health. Psychol..

[54] Cinciripini PM, Robinson JD, Karam-Hage M (2013). Effects of varenicline and bupropion sustained-release use plus intensive smoking cessation counseling on prolonged abstinence from smoking and on depression, negative affect, and other symptoms of nicotine withdrawal. JAMA Psychiatry..

[55] Kendzor DE, Businelle MS, Reitzel LR (2014). The influence of discrimination on smoking cessation among Latinos. Drug Alcohol. Depend..

[56] Cinciripini PM, Minnix JA, Green CE (2018). An RCT with the combination of varenicline and bupropion for smoking cessation. Clinical implications for front line use. Addiction..

[57] Fiore, M. C., Jaen, C. R., Baker, T. B., Bailey, W. C., Benowitz, N. L., Curry, S. J., Wewers, M. E. *Treating tobacco use and dependence: Treating Tobacco Use and Dependence: 2008 Update, Clinical Practice Guideline.* [Rockville, Md.]: U.S. Dept. of Health and Human Services, Public Health Service (2008).

[58] Cropsey KL, Trent LR, Clark CB, Stevens EN, Lahti AC, Hendricks PS (2014). How low should you go? Determining the optimal cutoff for exhaled carbon monoxide to confirm smoking abstinence when using cotinine as reference. Nicotine Tob. Res..

[59] Raiff BR, Faix C, Turturici M, Dallery J (2010). Breath carbon monoxide output is affected by speed of emptying the lungs. Implications for laboratory and smoking cessation research. Nicotine Tob. Res..

[60] Perkins KA, Karelitz JL, Jao NC (2013). Optimal carbon monoxide criteria to confirm 24-hr smoking abstinence. Nicotine Tob. Res..

[61] Li H, Durbin R (2010). Fast and accurate long-read alignment with Burrows-Wheeler transform. Bioinformatics..

[62] Challis D, Yu J, Evani US (2012). An integrative variant analysis suite for whole exome next-generation sequencing data. BMC Bioinf..

[63] Baylor College of Medicine. Human Genome Sequencing Center. https://www.hgsc.bcm.edu/software/cassandra. 10.1101/gr.8.3.170 9521920

[64] Illumina. *GenomeStudio.* San Diego, CA: Illumina Inc.

[65] Purcell S, Neale B, Todd-Brown K (2007). PLINK. A tool set for whole-genome association and population-based linkage analyses. Am. J. Hum. Genet..

[66] Pritchard JK, Stephens M, Donnelly P (2000). Inference of population structure using multilocus genotype data. Genetics..

[67] Alexander DH, Novembre J, Lange K (2009). Fast model-based estimation of ancestry in unrelated individuals. Genome Res..

[68] Alexander DH, Shringarpure SS, Novembre J, Lange K. Admixture 1.3 software manual. *Los Angeles: UCLA Human Genetics Software Distribution* (2015).

[69] Consortium 1GP. A map of human genome variation from population scale sequencing. *Nature. *467(7319), 1061 (2010). 10.1038/nature09534 PMC3042601 20981092

[70] Fairley S, Lowy-Gallego E, Perry E, Flicek P (2020). The international genome sample resource (IGSR) collection of open human genomic variation resources. Nucleic Acids Res..

[71] Rosenberg NA, Li LM, Ward R, Pritchard JK (2003). Informativeness of genetic markers for inference of ancestry. Am. J. Hum. Genet..

[72] Wu MC, Lee S, Cai T, Li Y, Boehnke M, Lin X (2011). Rare-variant association testing for sequencing data with the sequence kernel association test. Am. J. Hum. Genet..

[73] Lee S, Wu MC, Lin X (2012). Optimal tests for rare variant effects in sequencing association studies. Biostatistics..

[74] R Core Team. R: A Language and Environment for Statistical Computing. https://www.R-project.org. Accessed January 26 (2022).

[75] Manichaikul A, Mychaleckyj JC, Rich SS, Daly K, Sale M, Chen W-M (2010). Robust relationship inference in genome-wide association studies. Bioinformatics..

[76] Wang J, Shete S (2012). Testing departure from Hardy-Weinberg proportions. Methods Mol. Biol..

[77] Ingenuity Pathway Analysis. Ingenuity Pathway Analysis Software. www.ingenuity.com. Updated December 21 (2023).

[78] Muurling T, Stankovic KM (2014). Metabolomic and network analysis of pharmacotherapies for sensorineural hearing loss. Otol. Neurotol..

[79] Reyes-Gibby CC, Wang J, Yeung S-CJ, Shete S (2015). Informative gene network for chemotherapy-induced peripheral neuropathy. BioData Min..

[80] Ingenuity Systems. Ingenuity Pathways Analysis (IPA) of Large Datasets. http://www.usc.edu/hsc/nml/assets/bioinfo/IPA/Data%20Analysis%20training%20Handouts.pdf. Accessed May (2014).

[81] Reyes-Gibby CC, Wang J, Silvas MRT, Yu R, Yeung S-CJ, Shete S (2016). MAPK1/ERK2 as novel target genes for pain in head and neck cancer patients. BMC Genet..

[82] Ingenuity Systems. Calculating and interpreting the p‐values for functions, pathways, and lists in IPA (2010).

[83] Liu L, Chen X, Wang Y (2014). Notch3 is important for TGF-β-induced epithelial-mesenchymal transition in non-small cell lung cancer bone metastasis by regulating ZEB-1. Cancer Gene Ther..

[84] Merikallio H, Kaarteenaho R, Pääkkö P (2011). Zeb1 and twist are more commonly expressed in metastatic than primary lung tumours and show inverse associations with claudins. J. Clin. Pathol..

[85] Vu T, Jin L, Datta PK (2016). Effect of cigarette smoking on epithelial to mesenchymal transition (EMT) in Lung Cancer. J. Clin. Med..

[86] Cochet-Bissuel M, Lory P, Monteil A (2014). The sodium leak channel, NALCN, in health and disease. Front. Cell Neurosci..

[87] Sollis E, Mosaku A, Abid A (2023). The NHGRI-EBI GWAS Catalog. Knowledgebase and deposition resource. Nucleic Acids Res..

[88] Sahu A, Gopalakrishnan L, Gaur N (2018). The 5-Hydroxytryptamine signaling map. An overview of serotonin-serotonin receptor mediated signaling network. J. Cell Commun. Signal..

[89] Uhl GR, Drgon T, Johnson C, Ramoni MF, Behm FM, Rose JE (2010). Genome-wide association for smoking cessation success in a trial of precessation nicotine replacement. Mol. Med..

[90] M’e O, Zhou G, Li X (2018). The genetics of smoking in individuals with chronic obstructive pulmonary disease. Respir. Res..

[91] Kern SE, Kinzler KW, Bruskin A (1991). Identification of p53 as a sequence-specific DNA-binding protein. Science..

[92] Machiela MJ, Chanock SJ. LDlink. A web-based application for exploring population-specific haplotype structure and linking correlated alleles of possible functional variants. *Bioinformatics. *31(21), 3555–3557 (2015). 10.1093/bioinformatics/btv402. 10.1093/bioinformatics/btv402 PMC4626747 26139635

[93] RB1. https://www.ncbi.nlm.nih.gov/gene/5925.

[94] CDKN2A. https://www.ncbi.nlm.nih.gov/gene/1029.

[95] EGFR. https://www.ncbi.nlm.nih.gov/gene/1956.

[96] Nennig SE, Schank J (2017). The role of NFkB in drug addiction. Beyond inflammation. Alcohol Alcohol..

[97] Freathy RM, Mook-Kanamori DO, Sovio U (2010). Variants in ADCY5 and near CCNL1 are associated with fetal growth and birth weight. Nat. Genet..

[98] Roman TS, Cannon ME, Vadlamudi S (2017). A type 2 diabetes-associated functional regulatory variant in a pancreatic islet enhancer at the ADCY5 locus. Diabetes..

[99] Kim S-Y, Kim H-N, Jeon SW (2021). Association between genetic variants of the norepinephrine transporter gene (SLC6A2) and bipolar I disorder. Prog. Neuropsychopharmacol. Biol. Psychiatry..

[100] Angyal N, Horvath EZ, Tarnok Z (2018). Association analysis of norepinephrine transporter polymorphisms and methylphenidate response in ADHD patients. Prog. Neuropsychopharmacol. Biol. Psychiatry..

[101] Kim Y-K, Hwang J-A, Lee H-J (2014). Association between norepinephrine transporter gene (SLC6A2) polymorphisms and suicide in patients with major depressive disorder. J. Affect. Disord..

[102] Zhu AZX, Cox LS, Nollen N (2012). CYP2B6 and bupropion's smoking-cessation pharmacology. The role of hydroxybupropion. Clin. Pharmacol. Ther..

[103] Lerman C, Berrettini W (2003). Elucidating the role of genetic factors in smoking behavior and nicotine dependence. Am. J. Med. Genet. B Neuropsychiatr. Genet..

[104] Bloom J, Hinrichs AL, Wang JC (2011). The contribution of common CYP2A6 alleles to variation in nicotine metabolism among European-Americans. Pharmacogenet. Genomics..

[105] Bergen AW, McMahan CS, McGee S (2021). Multiethnic prediction of nicotine biomarkers and association with nicotine dependence. Nicotine & Tobacco Res..

[106] Costa R, Oliveira NG, Dinis-Oliveira RJ (2019). Pharmacokinetic and pharmacodynamic of bupropion. Integrative overview of relevant clinical and forensic aspects. Drug Metab. Rev..

[107] Yang Z, Seneviratne C, Wang S (2013). Serotonin transporter and receptor genes significantly impact nicotine dependence through genetic interactions in both European American and African American smokers. Drug Alcohol. Depend..

[108] Kim HW, Kang JI, Lee S-H (2016). Common variants of HTR3 genes are associated with obsessive-compulsive disorder and its phenotypic expression. Sci. Rep..

[109] Krzywkowski K, Davies PA, Feinberg-Zadek PL, Bräuner-Osborne H, Jensen AA (2008). High-frequency HTR3B variant associated with major depression dramatically augments the signaling of the human 5-HT3AB receptor. P. Natl. Acad. Sci. USA.

[110] Lipari, R.N., van Horn, S. *The CBHSQ Report: Smoking and Mental Illness among Adults in the United States.* Rockville (MD) (2013). 28459516

[111] Yuan S, Yao H, Larsson SC (2020). Associations of cigarette smoking with psychiatric disorders. Evidence from a two-sample Mendelian randomization study. Sci. Rep..

[112] Barkhuizen W, Dudbridge F, Ronald A (2021). Genetic overlap and causal associations between smoking behaviours and mental health. Sci. Rep..

[113] Ripke S, Wray NR, Lewis CM (2013). A mega-analysis of genome-wide association studies for major depressive disorder. Mol. Psychiatry..

[114] Mills EJ, Wu P, Lockhart I, Thorlund K, Puhan M, Ebbert JO (2012). Comparisons of high-dose and combination nicotine replacement therapy, varenicline, and bupropion for smoking cessation: a systematic review and multiple treatment meta-analysis. Ann. Med..

[115] Jimenez-Ruiz C, Berlin I, Hering T (2009). Varenicline: a novel pharmacotherapy for smoking cessation. Drugs..

[116] Lin Z, Walther D, Yu X-Y, Li S, Drgon T, Uhl GR (2005). SLC18A2 promoter haplotypes and identification of a novel protective factor against alcoholism. Hum. Mol. Genet..

[117] Randesi M, van den Brink W, Levran O (2019). VMAT2 gene (SLC18A2) variants associated with a greater risk for developing opioid dependence. Pharmacogenomics..

[118] Schwab SG, Franke PE, Hoefgen B (2005). Association of DNA polymorphisms in the synaptic vesicular amine transporter gene (SLC18A2) with alcohol and nicotine dependence. Neuropsychopharmacol..

[119] Sullivan PF, Neale BM, van den Oord E (2004). Candidate genes for nicotine dependence via linkage, epistasis, and bioinformatics. Am. J. Med. Genet. B Neuropsychiatr. Genet..

[120] Solovieff N, Roberts AL, Ratanatharathorn A (2014). Genetic association analysis of 300 genes identifies a risk haplotype in SLC18A2 for post-traumatic stress disorder in two independent samples. Neuropsychopharmacol..

[121] McClernon FJ, Kollins SH (2008). ADHD and smoking From genes to brain to behavior. Ann. NY Acad. Sci..

[122] Mace S, Taylor D (2000). Selective serotonin reuptake inhibitors. A review of efficacy and tolerability in depression. Exp. Opin. Pharmacother..

[123] David SP, Brown RA, Papandonatos GD (2007). Pharmacogenetic clinical trial of sustained-release bupropion for smoking cessation. Nicotine Tob. Res..

[124] Cinciripini PM, Tsoh JT, Wetter DW (2005). Combined effects of venlafaxine, nicotine replacement & brief counseling on smoking cessation. Exp. Clin. Psychopharmacol..

[125] Kaminskiy Y, Kuznetsova V, Kudriaeva A, Zmievskaya E, Bulatov E (2022). Neglected, yet significant role of FOXP1 in T-cell quiescence, differentiation and exhaustion. Front..

[126] National Library of Medicine National Center for Biotechnology Information. FOXP1 Forkhead Box P1. https://www.ncbi.nlm.nih.gov/gene/27086. Updated October 2022. Accessed November 18 (2022).

[127] Amos CI, Wu X, Broderick P (2008). Genome-wide association scan of tag SNPs identifies a susceptibility locus for lung cancer at 15q25.1. Nat. Genet..

[128] Thorgeirsson TE, Geller F, Sulem P (2008). A variant associated with nicotine dependence, lung cancer and peripheral arterial disease. Nature..

[129] Lips EH, Gaborieau V, McKay JD (2010). Association between a 15q25 gene variant, smoking quantity and tobacco-related cancers among 17 000 individuals. Int. J. Epidemiol..

[130] Truong T, Hung RJ, Amos CI (2010). Replication of lung cancer susceptibility loci at chromosomes 15q25, 5p15, and 6p21: A pooled analysis from the International Lung Cancer Consortium. J. Natl. Cancer Inst..

[131] Pillai SG, Ge D, Zhu G (2009). A genome-wide association study in chronic obstructive pulmonary disease (COPD) identification of two major susceptibility loci. PLoS Genet..

[132] Gabrielsen ME, Romundstad P, Langhammer A, Krokan HE, Skorpen F (2013). Association between a 15q25 gene variant nicotine-related habits lung cancer and COPD among 56 307 individuals from the HUNT study in Norway. Eur. J. Hum. Genet..

[133] Gibbons DL, Byers LA, Kurie JM (2014). Smoking, p53 mutation, and lung cancer. Mol. Cancer Res..

[134] Halvorsen AR, Silwal-Pandit L, Meza-Zepeda LA (2016). TP53 mutation spectrum in smokers and never smoking lung cancer patients. Front. Genet..

[135] Diakite B, Kassogue Y, Dolo G (2020). p.Arg72Pro polymorphism of P53 and breast cancer risk. A meta-analysis of case-control studies. BMC Med. Genet..

[136] Bories P, Prade N, Lagarde S (2020). Impact of TP53 mutations in acute myeloid leukemia patients treated with azacitidine. PLoS ONE..

[137] Khan MA, Tiwari D, Dongre A (2020). Exploring the p53 connection of cervical cancer pathogenesis involving north-east Indian patients. PLoS ONE..

[138] Piao J-M, Kim HN, Song H-R (2011). p53 codon 72 polymorphism and the risk of lung cancer in a Korean population. Lung Cancer..

[139] Toyooka S, Tsuda T, Gazdar AF (2003). The TP53 gene, tobacco exposure, and lung cancer. Hum. Mutat..

[140] Cardona AF, Rojas L, Zatarain-Barrón ZL (2019). Multigene mutation profiling and clinical characteristics of small-cell lung cancer in never-smokers vs. heavy smokers (Geno1.3-CLICaP). Front Oncol..

[141] Du X, Qi F, Lu S, Li Y, Han W (2018). Nicotine upregulates FGFR3 and RB1 expression and promotes non-small cell lung cancer cell proliferation and epithelial-to-mesenchymal transition via downregulation of miR-99b and miR-192. Biomed. Pharmacotherapy..

[142] El-Naggar AK, Lai S, Clayman G (1997). Methylation, a major mechanism of p16/CDKN2 gene inactivation in head and neck squamous carcinoma Am. J. Pathol..

[143] Asokan GS, Jeelani S, Gnanasundaram N (2014). Promoter hypermethylation profile of tumour suppressor genes in oral leukoplakia and oral squamous cell carcinoma. J. Clin. Diagn. Res.: JCDR..

[144] Marchetti A, Buttitta F, Pellegrini S (1997). Alterations of P16 (MTS1) in node-positive non-small cell lung carcinomas. J. Pathol.: J. Pathol. Soc. G.B. Irel..

[145] Huang T, Chen X, Hong Q (2015). Meta-analyses of gene methylation and smoking behavior in non-small cell lung cancer patients. Sci. Rep..

[146] MYC. https://www.ncbi.nlm.nih.gov/gene/4609.

[147] Lu L, Qi H, Luo F (2017). Feedback circuitry via let-7c between lncRNA CCAT1 and c-Myc is involved in cigarette smoke extract-induced malignant transformation of HBE cells. Oncotarget..

[148] Takeda K, Yamasaki A, Igishi T (2017). Frequency of Epidermal Growth Factor Receptor mutation in smokers with lung cancer without pulmonary emphysema. Anticancer Res..

[149] Ren J-H, He W-S, Yan G-L, Jin M, Yang K-Y, Wu G (2012). EGFR mutations in non-small-cell lung cancer among smokers and non-smokers A meta-analysis. Environ. Mol. Mutagenesis..

[150] Li H, Pan Y, Li Y (2013). Frequency of well-identified oncogenic driver mutations in lung adenocarcinoma of smokers varies with histological subtypes and graduated smoking dose. Lung Cancer..

[151] Gene Cards. DNAJC27 Gene - DnaJ Heat Shock Protein Family (Hsp40) Member C27. https://www.genecards.org/cgi-bin/carddisp.pl?gene=DNAJC27#function-gwas. Updated November 09, 2022. Accessed November 20 (2022).

[152] Gene Cards. EFR3B Gene—EFR3 Homolog B. https://www.genecards.org/cgi-bin/carddisp.pl?gene=EFR3B#diseases. Updated November 09, 2022. Accessed November 20 (2022).

[153] Taylor AE, Morris RW, Fluharty ME (2014). Stratification by smoking status reveals an association of CHRNA5-A3-B4 genotype with body mass index in never smokers. PLoS Genet..

