# Supplementary material for: Deep sequencing of candidate genes identified 14 variants associated with smoking abstinence in an ethnically diverse sample
Source: Sci Rep. 2024 Mar 16;14:6385. doi: 10.1038/s41598-024-56750-7 (PMC10944542; doi:10.1038/s41598-024-56750-7)

### Supplementary Materials

**Text S1. Ingenuity Pathway Analysis (IPA) Network Generation Algorithm**

In IPA, the nodes in a network represent molecules, including genes, chemicals, protein families, complexes, microRNA species, and biological processes, whereas lines (edges and arrows) connecting two molecules represent relationships between them. The network is created using the focus genes, which, in our study, are the genes that harbor the genetic variants identified to be significantly associated with the abstinence phenotype in the association analysis. The key hypothesis in developing a network in the IPA core analyses is that the biological function involves locally dense interactions. The network generation algorithm comprises the following steps:

(1) Rank the focus genes in a decreasing order based on their triangular connectivity, which measures the number of triangular connections in which a gene functions (or pairs of genes to which a gene is connected);

(2) Use the most connected focus gene as the starting seed gene and generate a seed gene network by incorporating a subset of the remaining focus genes situated in the neighborhood of the starting seed gene. Here, a neighborhood is defined as a gene plus the genes exactly one connection away from it;

(3) Generate the second seed gene network using the focus genes not included in the first seed gene network. Repeat this process until all focus genes are presented in a relevant seed gene network;

(4) Connect the small seed gene networks through additional non-focus genes to generate larger networks;

(5) Connect additional genes or networks from IPA’s database to the existing network if the network has not reached the maximum pre-specified network size (e.g., 140 genes). In this process, IPA prioritizes genes with the largest overlap with the existing network and the fewest number of neighbors, measured using a metric called specific connectivity. Specific connectivity is calculated by dividing the number of genes in the intersection of the neighborhood and the existing network by the union of the number of genes in the neighborhood and the existing network. The gene with the highest specific connectivity score is included in the existing network.

Using this network generation algorithm, the IPA analysis attempts to connect additional non-focus genes from its entire database to any of the genes which have already involved in the gene network (focus or non-focus genes) if such genes are more likely to have connections (i.e., biological relationships) with the network.

**Table S1. List of candidate genes used for sequencing and genotyping**

| **Candidate Genes** | |
| --- | --- |
|  |  |
| *ADCY1* | *CHRNB2* |
| *ADCY2* | *CHRNB3* |
| *ADCY3* | *CHRNB4* |
| *ADCY4* | *CHRND* |
| *ADCY5* | *CHRNG* |
| *ADCY6* | *CREB1* |
| *ADCY7* | *CYP2A13* |
| *ADCY8* | *CYP2A6* |
| *ADRA1A* | *CYP2A7* |
| *ADRA1B* | *CYP2B6* |
| *ADRA1D* | *CYP2B7P1* |
| *ADRA2A* | *CYP2E1* |
| *ADRA2B* | *CYP2G1P* |
| *ADRA2C* | *DBH* |
| *ADRB1* | *DRD1* |
| *ADRB2* | *DRD2* |
| *ARRB2* | *DRD3* |
| *CDK5R1* | *DRD4* |
| *CHAT* | *HTR3A* |
| *CHRFAM7* | *HTR3B* |
| *CHRNA1* | *HYKK* |
| *CHRNA2* | *IREB2* |
| *CHRNA3* | *PSMA4* |
| *CHRNA4* | *SLC22A2* |
| *CHRNA5* | *SLC6A2* |
| *CHRNA6* | *SLC6A3* |
| *CHRNA7* |  |
| *CHRNA9* |  |

**Table S2. Linkage disequilibrium between SNPs showed association with smoking cessation in literature and 14 SNPs identified in the association analysis.**

| **SNPs**  **from literature** | **SNPs**  **from our study** |  | ***r^2^*** | **Comment** |
| --- | --- | --- | --- | --- |
|  |  |  |  |  |
| rs7159300 | rs1175607105 |  | - | rs1175607105 Variant is not in 1000G reference panel. |
| rs7159300 | rs2173763 |  | 0.010 | rs7159300 and rs2173763 are on different chromosomes. |
| rs7159300 | rs6749438 |  | 0.005 | rs7159300 and rs6749438 are on different chromosomes. |
| rs7159300 | rs6718083 |  | <0.001 | rs7159300 and rs6718083 are on different chromosomes. |
| rs7159300 | rs7349 |  | 0.018 | rs7159300 and rs7349 are on different chromosomes. |
| rs7159300 | rs6869603 |  | 0.016 | rs7159300 and rs6869603 are on different chromosomes. |
| rs7159300 | rs363222 |  | 0.002 | rs7159300 and rs363222 are on different chromosomes. |
| rs7159300 | rs1413172952 |  | - | rs1413172952 Variant is not in 1000G reference panel. |
| rs7159300 | rs1288980 |  | 0.010 | rs7159300 and rs1288980 are on different chromosomes. |
| rs7159300 | rs1204720503 |  | - | rs1204720503 Variant is not in 1000G reference panel. |
| rs7159300 | rs992528 |  | 0.014 | rs7159300 and rs992528 are on different chromosomes. |
| rs7159300 | rs80210037 |  | - | rs80210037 Variant is not in 1000G reference panel. |
| rs7159300 | rs11064432 |  | 0.018 | rs7159300 and rs11064432 are on different chromosomes |
| rs7159300 | rs1333758 |  | 0.011 | rs7159300 and rs1333758 are on different chromosomes. |
|  |  |  |  |  |
| rs207675 | rs1175607105 |  | - | rs1175607105 Variant is not in 1000G reference panel. |
| rs207675 | rs2173763 |  | 0.008 | rs207675 and rs2173763 are on different chromosomes. |
| rs207675 | rs6749438 |  | 0.004 | rs207675 and rs6749438 are on different chromosomes. |
| rs207675 | rs6718083 |  | 0.001 | rs207675 and rs6718083 are on different chromosomes. |
| rs207675 | rs7349 |  | 0.026 | rs207675 and rs7349 are on the same chromosome. |
| rs207675 | rs6869603 |  | 0.014 | rs207675 and rs6869603 are on different chromosomes. |
| rs207675 | rs363222 |  | 0.015 | rs207675 and rs363222 are on the same chromosome. |
| rs207675 | rs1413172952 |  | - | rs1413172952 Variant is not in 1000G reference panel. |
| rs207675 | rs1288980 |  | 0.021 | rs207675 and rs1288980 are on different chromosomes. |
| rs207675 | rs1204720503 |  | - | rs1204720503 Variant is not in 1000G reference panel. |
| rs207675 | rs992528 |  | 0.013 | rs207675 and rs992528 are on the same chromosome. |
| rs207675 | rs80210037 |  | - | rs80210037 Variant is not in 1000G reference panel. |
| rs207675 | rs11064432 |  | 0.024 | rs207675 and rs11064432 are on different chromosomes. |
| rs207675 | rs1333758 |  | 0.011 | rs207675 and rs1333758 are on different chromosomes. |
|  |  |  |  |  |
| rs212420 | rs1175607105 |  | - | rs1175607105 Variant is not in 1000G reference panel. |
| rs212420 | rs2173763 |  | 0.008 | rs212420 and rs2173763 are on different chromosomes. |
| rs212420 | rs6749438 |  | 0.004 | rs212420 and rs6749438 are on different chromosomes. |
| rs212420 | rs6718083 |  | 0.001 | rs212420 and rs6718083 are on different chromosomes. |
| rs212420 | rs7349 |  | 0.027 | rs212420 and rs7349 are on the same chromosome. |
| rs212420 | rs6869603 |  | 0.014 | rs212420 and rs6869603 are on different chromosomes. |
| rs212420 | rs363222 |  | 0.015 | rs212420 and rs363222 are on the same chromosome. |
| rs212420 | rs1413172952 |  | - | rs1413172952 Variant is not in 1000G reference panel. |
| rs212420 | rs1288980 |  | 0.021 | rs212420 and rs1288980 are on different chromosomes. |
| rs212420 | rs1204720503 |  | - | rs1204720503 Variant is not in 1000G reference panel. |
| rs212420 | rs992528 |  | 0.013 | rs212420 and rs992528 are on the same chromosome. |
| rs212420 | rs80210037 |  | - | rs80210037 Variant is not in 1000G reference panel. |
| rs212420 | rs11064432 |  | 0.024 | rs212420 and rs11064432 are on different chromosomes. |
| rs212420 | rs1333758 |  | 0.011 | rs212420 and rs1333758 are on different chromosomes. |

**Figure S1. Manhattan plot for the association study of smoking abstinence phenotype in joint analysis.**

**
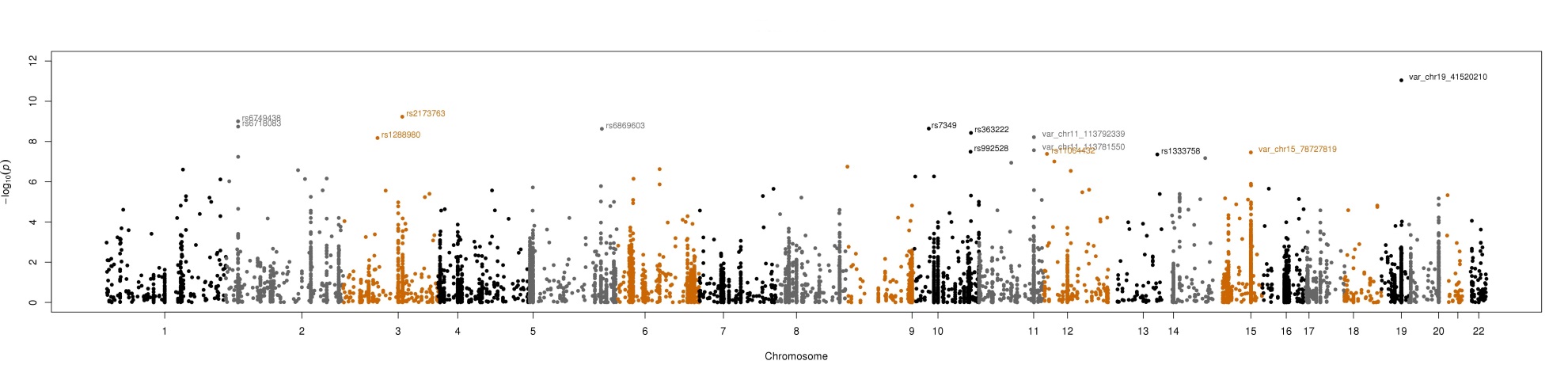
**

**Figure S2. The network generated by IPA core function analysis for smoking cessation using 10 focus genes identified in the association analyses (Table 2). Green nodes: focus genes; red nodes: molecules with ≥ 15 connections. Dashed and solid lines represent indirect and direct interactions, respectively.**


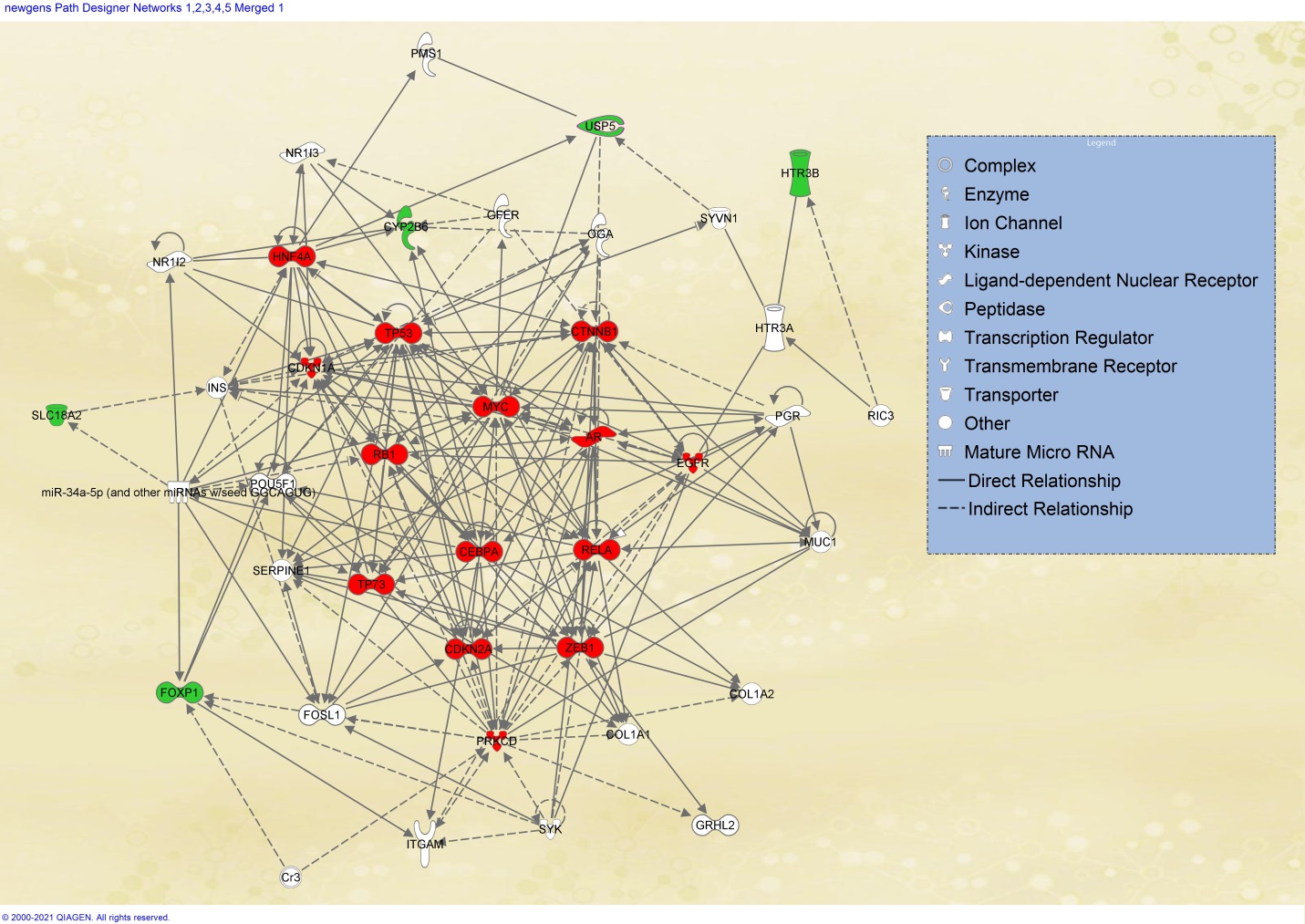

Supplement: Supplementary file 2 — Supplementary Information 2. [file 41598_2024_56750_MOESM2_ESM.docx]
